# Transglutaminases (TGs) in Ocular and Periocular Tissues: Effect of Muscarinic Agents on TGs in Scleral Fibroblasts

**DOI:** 10.1371/journal.pone.0018326

**Published:** 2011-04-06

**Authors:** V. A. Barathi, Sung R. Weon, Queenie S. W. Tan, Kwan J. Lin, Louis Tong, Roger W. Beuerman

**Affiliations:** 1 Ocular Disease Model Research Group, Singapore Eye Research Institute, Singapore, Singapore; 2 Singapore National Eye Centre, Singapore, Singapore; 3 SRP Neuroscience and Behavioral Disorder, DUKE-NUS Graduate Medical School, Singapore, Singapore; 4 Department of Ophthalmology, Yong Loo Lin School of Medicine, National University of Health Sciences, National University Hospital, Singapore, Singapore; Alcon Research, Ltd., United States of America

## Abstract

**Objective:**

To investigate the expression of transglutaminases (TGs) in the ocular surface, the eyelid margin and associated glands and to determine effect of muscarinic agents on TGs in scleral fibroblasts (SF).

**Materials and Methods:**

Primary SFs cultured from mouse and human sclera were treated with atropine and carbachol for 5 days. Lysed cell RNA was used for real-time PCR, protein was used for Western blot analysis and TG-2 transamidase activity was measured by ELISA. Immunohistochemistry was done to determine the expression of TGases.

**Results:**

Immunohistochemistry and western blot confirmed the expression of TGs-1, 2, 3 and 5 proteins in cultured SFs and eye tissues. Real time PCR showed TG-1, 2, 5 transcript levels to be down regulated 3 fold (p<0.05) in cultured human and mouse SFs after incubation with atropine and this was reversed by carbachol. However, TG-3 expression was increased with atropine and decreased with carbachol at all concentrations. Atropine abrogated the carbachol-induced activation of SF in a dose-dependent manner. TGs-1, 3, 5 were localized in the entire mouse corneal epithelium, stroma and endothelium but TG-2 was present only in the corneal subepithelium and stroma. All TGs were localized in mouse Meibomian glands however TG-2 had a weak expression.

**Conclusions:**

Our results confirm that TGs-1, 2, 3 and 5 are expressed in human SF and murine ocular tissues, eyelid and associated Meibomian glands. Real-time PCR and Western blot results showed that muscarinic antagonist down-regulates TGs-1, 2 and 5 in both cultured human and mouse SFs and upregulates TG-3. Atropine abrogated the carbachol-induced activation of SF in a dose-dependent manner. These results suggest that manipulation of TGs by way of muscarinic receptor acting drugs may be a plausible method of intervention in wound healing and scleral remodeling.

## Introduction

Transglutaminases (TGs) are a big class of intra- and extracellular enzymes with at least 8 members, all of which catalyze the formation of epsilon - (γ-glutamyl) lysine isopeptide linkages between peptide substrates. These enzymes are tightly regulated, and are involved in processes such as inflammation, re-epithelialization, neovascularization, synthesis and stabilization of a fibrous extracellular matrix (ECM) [1]–[3].

Different types of TGs are found in various cellular compartments. TG-1 is located in the cytosolic and membrane compartments only whereas TG-2 is present in cell nucleus in addition to cytosolic and membrane compartment. TGs-3 and 5 are restricted to the cytosolic compartment. Certain forms of TGs, TG-4 and Factor XIII are extracellular moieties [1]. Since TGs are found in different sub-cellular locations, it is not surprising that they also sub-serve different functions in these locations.

Cell adhesion and cell spreading are integral functions regulated by TGs. Primary fibroblasts from TG-2 knock out mice have decreased adherence to culture vessels [4]. There are a few ways that TG-2 may be involved in remodeling of ECM. First, TG-2 may affect the covalent cross-linking of ECM molecules in the extracellular space, hence affecting stabilization and degradation of these molecules [5], [6]. Second, TG-2 may affect the motility, adhesion and survival of the ECM producing fibroblasts, hence influencing the amount of connective tissue molecules produced [7], [8]. Finally, TG-2 can release inactive precursors of molecules that remodel the matrix sequestered in the matrix [9], [10].

Wound healing is a dynamic process with different temporal phases. By modifying the activity of extracellular matrix proteases, wound healing in the damaged ocular surface may proceed in a favorable or unfavorable manner, depending on the timing and duration of intervention. Apoptosis in the ocular surface is not only present in an experimental model of dry eye [11], but contributes significantly to the disease process by reducing tear secreting components such as Goblet cells and accessory lacrimal glands.

TGs are involved in wound healing diseases such as pterygium [12], allergic conjunctivitis [13], dry eye [14], cicatricial conjunctivitis [15], [16] and also in glaucoma [9], [17]. In the cornea and conjunctiva, TGs activity can be detected in the intercellular spaces, along the basement membranes, cytoplasm of the epithelial cells, superficial stromal keratocytes, as well as in the walls of conjunctival stromal vessels [18]. In the sclera, a previous study indicated that TG activity was localized to mainly to the episcleral vessel walls [18]. The only TG that is well studied in ocular surface diseases, TGase-1 was up-regulated in cicatrizing diseases and dry eye [15], [16]. Although ocular tissue and associated glands are well innervated by muscarinic cholinergic motor neurons, and carbachol a muscarinic agonist [19] can increase TG mediated transamidation. The molecular mechanism of activation and signaling pathway upstream of TGs has not yet been elucidated in ocular surface tissues.

As a first step to understand the importance of TGs in critical processes such as wound healing and modulation of inflammation in ocular diseases, we aimed to investigate the expression of TGs in the ocular surface, the eyelid margin, associated glands and their regulation by muscarinic receptor acting drugs. In addition we aimed to study the effects of muscarinic receptor signaling on TGs expression in cultured cells. These expression and functional studies will likely shed light on the pathological conditions such as ocular surface wound healing, pterygium, cataract, glaucoma, myopia and proliferative vitreoretinopathy.

## Materials and Methods

### Human and Animal Tissues

Human scleral tissues (*n = 6*) harvested within 24 hours from normal cadaver eyes (age range, 45–80 years) obtained at autopsy were provided by the Singapore Eye Bank. The protocol was approved by the Institutional Review Board of the Singapore Eye Research Institute. All study procedures were performed as part of standard clinical care and complied with the tenets of the Declaration of Helsinki regarding human research, and as all procedures performed were essential for standard clinical care of these patients, written consent was not required, but consent was obtained by assent. The patient's next of kin were aware of the privacy policy of the hospital that states that information released for publication would not include patient identifiers.

Mice (Balb/c, n = 60) were obtained from the animal holding unit of the National University of Singapore. Approval was obtained from the Singhealth Institutional Animal Care and Use of Committee (IACUC; AALAC accredited, #2008/SHS/359) and all aspects of the study were in accordance with the Association for Research in Vision and Ophthalmology (ARVO) recommendations for animal experimentation.

### Primary Cell culture and drug treatment

Fibrous sclera (n = 3 batches; 30 sclera/batch) was placed in 60 mm culture dish with dubelcco's Modified Eagle's Medium (DMEM, Invitrogen-Gibco, Grand Island, NE) supplemented with penicillin, streptomycin and amphotericin B and 10% Fetal Bovine Serum (FBS, Invitrogen-Gibco). Tissue culture were incubated at 37°C, 5% CO_2_ and allowed to reach 80% confluence. Cells were passaged sequentially by exposing cells to 0.25% Trypsin/0.5 mM EDTA at 37°C for 5 minutes. All cells used in experiments were below passages 3.

Passaged cells were plated at a concentration of 1×10^5^ into 6 well plates containing DMEM with 10% FBS. The cells were seen to attach to the bottom of the culture wells after 4 hours. Freshly prepared atropine and carbachol at final 0.01, 0.1, 1, 5 and 10 µM concentrations were added for 5 days. To study the combined effect of atropine and carbachol, SFs were pre-treated with atropine (0.01, 0.1, 1, 5 and 10 µM) 4 hours before being incubated with equimolar carbachol. The culture medium was removed and fresh medium containing the same muscarinic drug added every day to avoid drug changes such as atropine oxidation. Cell-lysate was collected for relative quantitative (q) PCR and protein analysis at day 5.

### Sample preparation

The cells (n = 3 sets; 1×10^6^ cells/set) were lysed by sonification in 1× RIPA lysis buffer (Santa Cruz Biotechnology, Santa Cruz, CA) with 10 µl PMSF solution, 10 µl sodium orthovanadate solution and 20 µl protease inhibitor cocktail solution. After centrifugation at 20,000 ×*g* at 4°C for 20 minutes, and the supernatant collected. The protein content in the supernatants was measured using the DC Protein Assay kit (Bio-Rad, Berkeley, CA) following the manufacturer's instructions. Samples were stored at −80°C until assayed.

### Immunohistochemistry and Immunocytochemistry

The whole mouse eye, eyelid (2 months old, n = 6) and human sclera (n = 3) were embedded in OCT (frozen tissue matrix) compound at −20°C for 1 hour. Prepared tissue blocks were sectioned with cryostat at 5 microns thicknesses and collected on clean polysine™ glass slides. Sections were fixed with 4% para-formaldehyde for 10 minutes. After washing 3× with 1× PBS for 5 minutes, 4% goat serum diluted with 1× PBS was added as a blocking buffer. The slides were then covered and incubated for 1 hour at room temperature (RT) in a humid chamber. After rinsing with 1× PBS, a specific primary antibody for TGs-1, 2, 3 and 5 (polyclonal raised in rabbit, Abcam, Cambridge, UK) diluted (1∶100) with 2% goat serum was added and incubated further at 4°C in a humid chamber overnight. After washing 3× with 1× PBS for 10 minutes, fluorescein-labeled goat anti-rabbit secondary antibody (1∶200, Chemicon International, Temecula, CA) was applied and incubated for 90 minutes at RT. After washing and air-drying, slides were mounted with antifade medium containing DAPI (4, 6-diamidino-2-phenylindole; Vectashield; Vector Laboratories, Burlingame, CA) to visualize the cell nuclei. Sections incubated with 2% goat serum with omitted primary antibody were used as a control.

Fresh human and mouse SF cells were cultured on sterile chamber slides (n = 6). Cells were washed with phosphate buffered saline (PBS) and fixed with ice-cold methanol: acetone (1∶1) at −20 for 10 minutes and air-dried. Cells were permeabilised with 0.5% Triton-X 100 in PBS for 2 minutes at RT. Non-specific sites were blocked with 1% bovine serum albumin (BSA), 0.3% Triton X-100 in PBS for 30 minutes at RT. The cells were then processed and stained as described before. A fluorescence microscope (Axioplan2; Carl Zeiss Meditec, GmbH, Oberkochen, Germany) was used to capture images. Experiments were repeated in triplicates from 3 different samples.

### Electrophoresis and Immunoblotting

Proteins in the supernatant were separated by SDS-PAGE, transferred to nitrocellulose membranes, blocked in 5% BSA in TBST (10 mM Tris-HCl [pH 8.0], 150 mM NaCl, and 0.0.05% Tween-20) for 2 hours at RT, and incubated with the same anti-TGs antibodies described above at a dilution of 1∶1000 and anti β-tubulin antibodies used as a loading control, for 1 hour at RT. The membranes were washed 3 times in TBST and incubated with HRP-conjugated secondary antibody (Chemicon International) at a dilution of 1∶2500 for 1 hour at RT. Immunoreactive bands were visualized using the enhanced chemiluminescence method (GE Healthcare, Buckinghamshire, UK). The membrane was wrapped in plastic and placed against an X-ray film to expose for an appropriate length of time (30 seconds-5 minutes).

### Transglutaminase Activity Assay

This assay was designed to assess the cross-linking activity of TG-2 between two proteins. TG-2 activity was measured with Transglutaminase colorimetric Microassay kit (TG-Covtest, Covalab, Cambridge, UK). The approximate volume of reagents to use, the wavelengths to use to read absorbance, the incubation times were determined from previous study that have carried experiments with similar principles [20], [21]. In order to standardize the protein concentration to use from the samples obtained, we used 100 µg protein per well after several repeats with different protein concentrations. Control and samples were subjected to exactly the same assay procedure. All reagents used and protein coated plates were part of the kit. Biotin cadaverine was reconstituted with 6 ml of deionized water and 90 µl was added into each well of the 96 well plate that comes coated with CBZ-GLN-GLY. The rest of the steps were followed as in the manufacturer's instructions and finally absorbance was read at 450 nm using a microplate reader (TECAN, Austria). All incubations were performed with gentle shaking on a laboratory orbital shaker.

### Quantitative Real-Time Comparative PCR

Total RNA was isolated from cultured human and mouse SFs (n = 3 sets; 1×10^6^ cells/set) using TRIzol reagent (Invitrogen life technologies, USA) in accordance with the manufacturer's instructions [22]. Genomic DNA was removed by digestion with DNase I (Amp Grade; Invitrogen-Gibco) for 15 minutes at room temperature. One microgram of total RNA was reverse-transcribed with random hexamers by using a first-strand cDNA synthesis kit (Invitrogen-Gibco). qPCR was performed in a 384-well plate format on an Roche 480 LightCycler Detection System (Roche Applied Science, Mannheim, Germany) with efficiency corrected software 4.0. PCR was performed using 50 ng of cDNA of each sample. The pre-validated hydrolysis probes for TGs-1, 2, 3 and 5 were from human and mouse universal probe library (Roche) and the primers for human and mouse are shown in Table 1 and 2 respectively. GAPDH Internal Standard (Roche) was used as an endogenous control. To standardize and evaluate scleral gene expression, aliquots of the same cDNA (50 ng) preparation were used as templates in all PCR reactions. The data was analyzed by comparative *C*_T_ (ΔΔ*C*_T_) method as previously described [23].

**Table 1:** Human TGM probes and primers for qPCR reactions.

Gene	Primers	Sizes	Prevalidated Probes
TGM 1	5′ CCC CAA GAG ACT AGC AGT GG 3′ (Fw)5′ AGG CCA TTC TTG ATG GAC - 3′ (Rev)	84 bp	# 16
TGM 2	5′ AGG GTG ACA AGA GCG AGA TG - 3′ (Fw)5′ TGG TCA TTC ACG ACT CCA C – 3′ (Rev)	96 bp	# 32
TGM 3	5′ TGG AAG GAC TCT GCC ACA AT - 3′ (Fw)5′ TGA GCG TAC GAG ATC TTT ATG G -3′ (Rev)	137 bp	# 21
TGM 5	5′ GGG GAG TTC ATC CTG CTT TT -3′ (Fw)5′ CTG GGG TTC ACT GTC CAA GT -3′ (Rev)	191 bp	# 20

**Table 2:** Mouse TGM probes and primers for qPCR reactions.

Gene	Primers	Sizes	Prevalidated Probes
TGM 1	5′ GCC CTT GAG CTC CTC ATT G 3′ (Fw)5′ CCC TTA CCC ACT GGG ATG AT - 3′ (Rev)	74 bp	# 10
TGM 2	5′ GGT GAT CCT CGC TTG AGT GT - 3′ (Fw)5′ CTC CAA ATC ACA CCT CTC CAG – 3′ (Rev)	94 bp	# 17
TGM 3	5′ GAT CAC AGC TGT TTG CAA GG - 3′ (Fw)5′ CAT GAG CCT GTT CCA GCA C -3′ (Rev)	107 bp	# 31
TGM 5	5′ TCC ATC CAG CTG TCT GTG G -3′ (Fw)5′ AGG GCC ACC TCT AGT CCT GT -3′ (Rev)	91 bp	# 26

### Data Analysis

Statistical comparisons between experimental groups were conducted using Student's t-test or one-way ANOVA (Statistica 6.0, SPSS, Chicago, IL, USA). The Mann-Whitney U-test was used to determine differences between groups. A significance level of p<0.05 was used. Data are presented as means ± standard deviation.

## Results

### Expression of TGs in the Ocular Surface and associated glands

**Figure 1:**
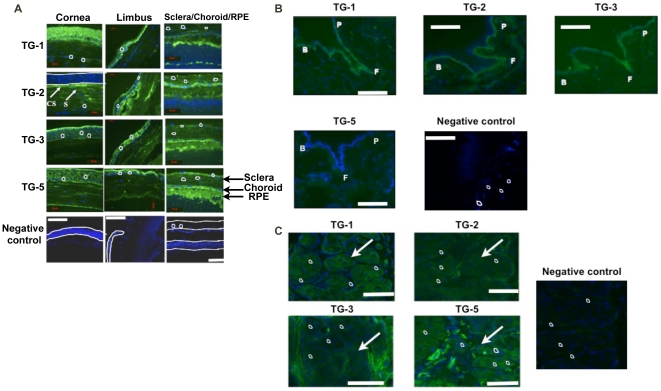
Localization of transglutaminases (TGs) in mouse eye. (A) To determine the presence of TGs in the mouse eye, the whole eye sections (5 microns) were stained using anti mouse TG-1, TG-2, TG-3 and TG-5 rabbit IgG-fluorescein conjugates. The negative control section was incubated with 2% goat serum without the respective primary antibodies. Positive immunostaining of TGs were identified in the mouse cornea, retina pigment epithelium (RPE), choroid and sclera, as compared to the negative control. The localization of TGase-2 in cornea is different from the other 3 TGs. TG-1, TG-3, TG-5 were localized in the entire mouse corneal epithelium, stroma and endothelium but TG-2 was present only in the corneal subepithelium (“CS”) and stroma (“S) (see white arrows). Error bar = 50 µM. DAPI stains nuclei (indicated by the white circles and the white boundaries) and FITC stains cell membrane and cytoplasm. Magnification at 200×. (B) shows the localization of TG-1, TG-2, TG-3 in the mouse palpebral (P), forniceal (F) and bulbar (B) conjunctiva but not TG-5, by immunofluorescent staining. Error bar = 50 µM. DAPI stains nuclei (indicated by white circles) and FITC stains cell membrane and cytoplasm. Magnification at 200×. (C) shows the localization of TGs in mouse meibomian glands. All TGs were expressed in mouse meibomian glands but TG-2 was weakly detected. Error bar = 50 µM. Arrow indicates the meibomian gland. DAPI stains nuclei (indicated by white circles) and FITC stains cell membrane and cytoplasm. Magnification at 200×.

Positive immunostaining of TGs was shown in the mouse eye tissues (Figure 1A). This finding is useful for our understanding of the gene expression of TGs in mouse eye development. TGs-1, 3, 5 were localized in the entire mouse corneal epithelium, stroma and endothelium but TG-2 was present only in the corneal subepithelium and stroma. All TGs showed staining in the RPE, choroid and sclera, with scleral staining between the collagen fibre bundles. No immunostaining was observed in the sections incubated with 2% goat serum and DAPI stains the nucleus. Immunofluorescent staining showed the localization of TGs-1, 2, 3 in the mouse palpebral, forniceal and bulbar conjunctiva but not TG-5 (Figure 1B). All these TGs were expressed in mouse meibomian glands (Figure 1C) but TG-2 was weakly detected.

**Figure 2:**
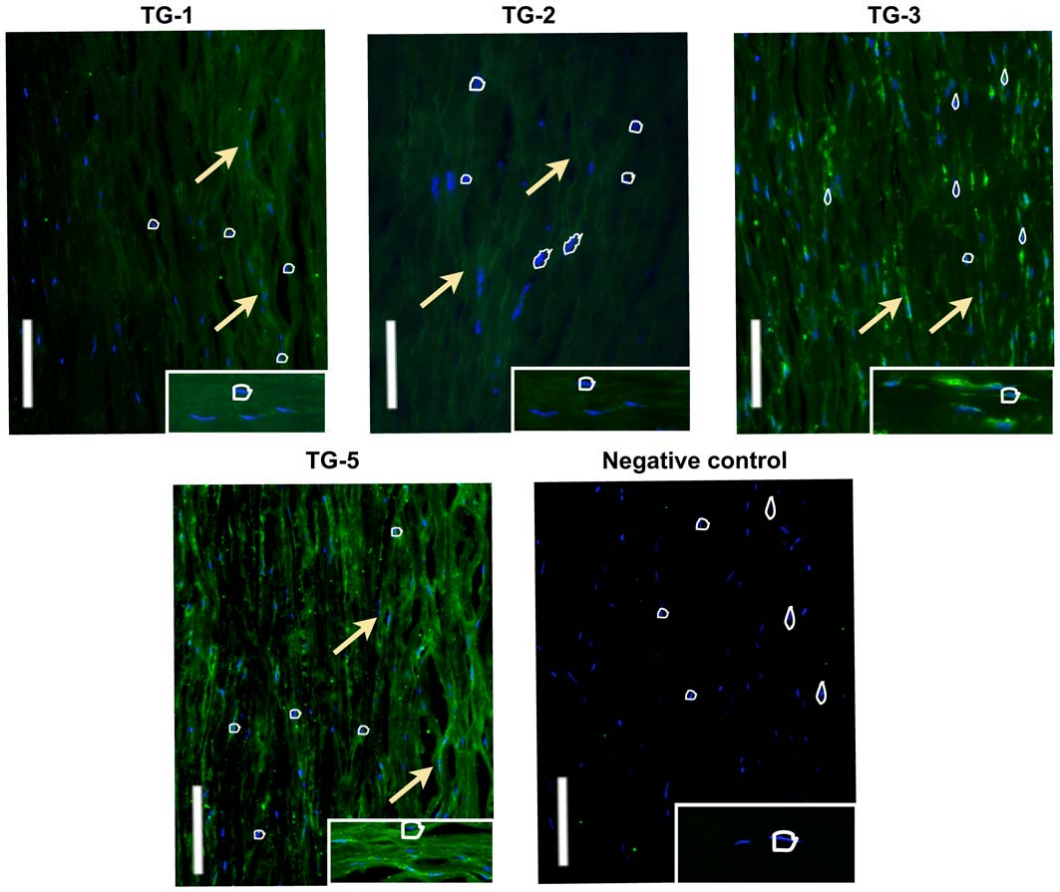
Localization of TGs in human sclera. The localization of TG-1, TG-2, TG-3 and TG-5 in the human scleral tissue with scleral staining between the collagen fibre bundles was determined by immunofluorescent staining. No immunostaining was found in the negative control. Error bar = 50 µM. Arrow indicates the scleral fibroblasts. DAPI stains nuclei (indicated by the white circles) and FITC stains cell membrane and cytoplasm. Magnification at 200×.

**Figure 3:**
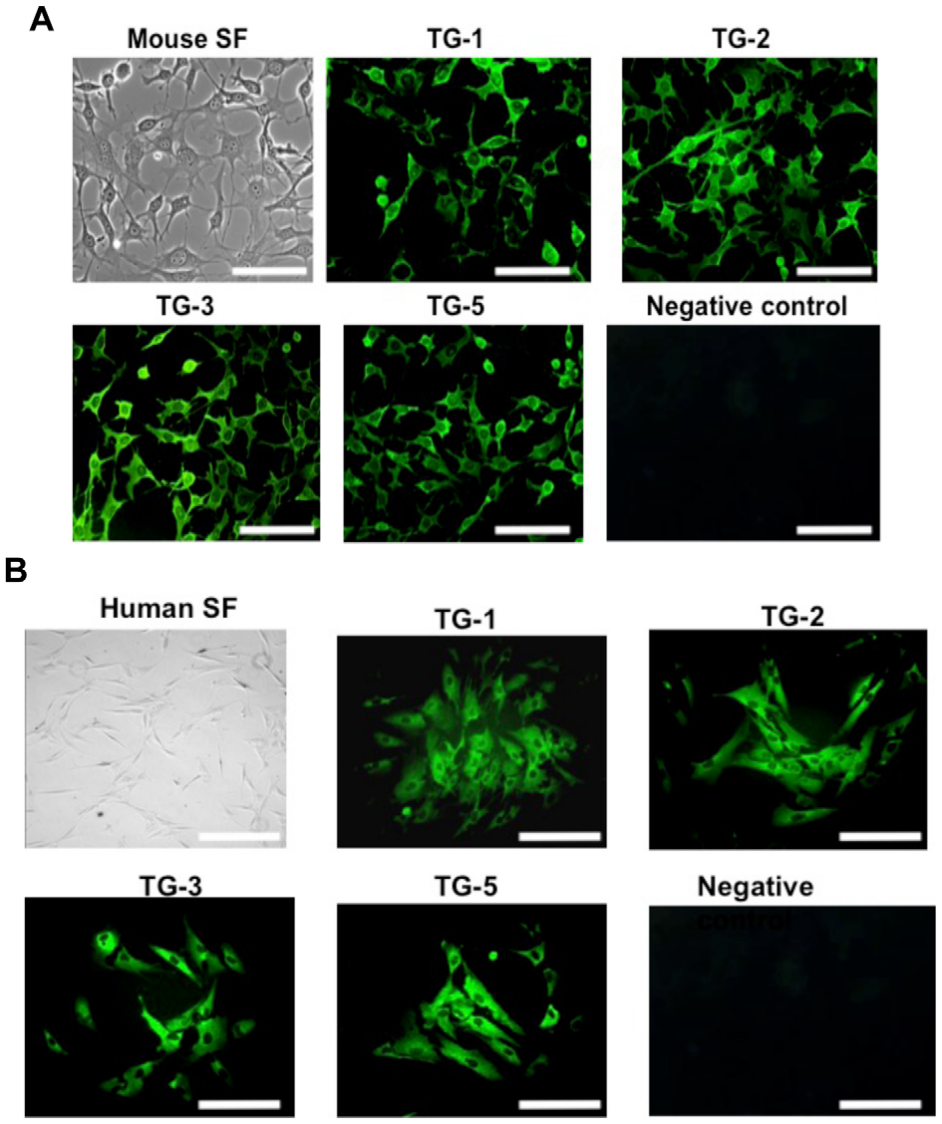
Expression of TGs in mouse and human scleral fibroblasts. (A) The cultured mouse scleral fibroblasts expressed all 4 TGs at cellular level. TG-1, TG-3 and TG-5 were located in the cytosolic and membrane compartments only whereas TG-2 was present in cell nucleus along with cytosolic and membrane compartment. Error bar = 50 µM. Magnification at 200×. (B) The cultured human scleral fibroblasts expressed all 4 TGs at cellular level. TG-1, TG-3 and TG-5 were located in the cytosolic and membrane compartments only whereas TG-2 was present in cell nucleus along with cytosolic and membrane compartment. Error bar = 50 µM. Magnification at 200×.

Positive immunostaining of TGs was shown in the human scleral tissues (Figure 2). Primary cultured mouse and human SFs expressed all 4 TGs (Figure 3A and B respectively). TGs-1, 3 and 5 were located in the cytosolic and membrane compartments only whereas TG-2 was present in cell nucleus along with cytosolic and membrane compartment.

### Confirmation of TG proteins in mouse and human scleral fibroblasts

**Figure 4:**
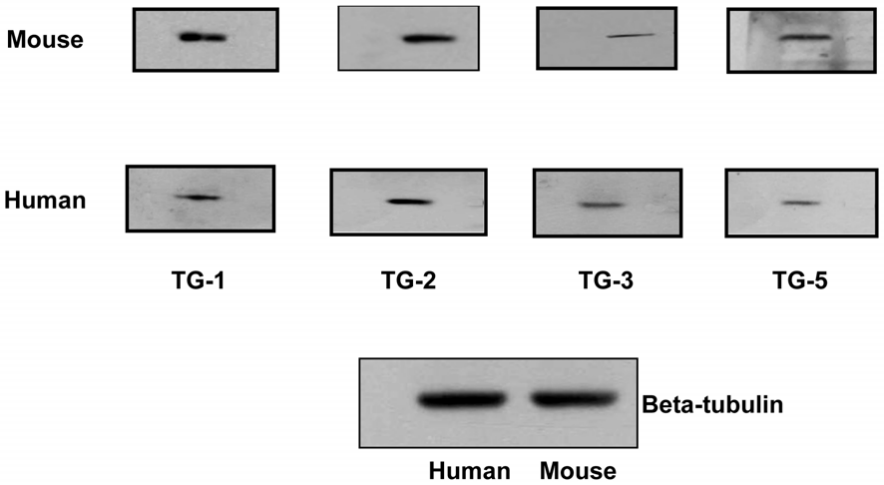
Confirmation of TG proteins by Western blot analysis. Western blot confirmed the expression of TG-1, TG-2, TG-3 and TG-5 proteins in mouse and human scleral fibroblasts. ß-tubulin was used as a loading control. TG-1: 90 kDa, TG-2: 82 kDa, TG-3: 77 kDa, TG-5: 80 kDa and ß-tubulin (loading control): 55 kDa.

Western blot was carried out in the cultured mouse and human SF to confirm the expression of TGs at protein level and ß-tubulin was used as a loading control (Figure 4).

### Effect of TG proteins upon muscarinic agents stimulation

**Figure 5:**
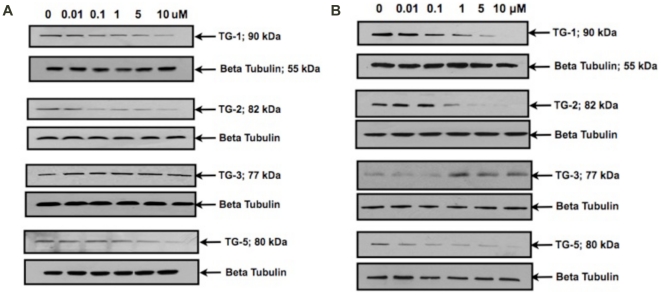
Effect of atropine treatment on TGs in mouse and human scleral fibroblasts. (A) Western blot image detecting TG proteins in mouse scleral fibroblasts after atropine treatment. P2 cultured mouse scleral fibroblasts were treated with atropine at concentrations of 0.01 µM, 0.1 µM, 1 µM, 5 µM and 10 µM for 5 days. Following 5 days of treatment, the total cellular protein was extracted from these cells and TG proteins detected via Western blot analysis. It can be observed that the TG-1, 2 and 5 protein levels were reduced after atropine treatment. However, TG-3 protein level was increased after receiving atropine. ß-tubulin was used as a loading control. (B) Western blot image detecting TG proteins in human scleral fibroblasts after atropine treatment. P2 cultured human scleral fibroblasts were treated with atropine at concentrations of 0.01 µM, 0.1 µM, 1 µM, 5 µM and 10 µM for 5 days. Following 5 days of treatment, the total cellular protein was extracted from these cells and TG proteins detected via Western Blot analysis. It can be observed that the TG-1, 2 and 5 protein levels were reduced after atropine treatment. However, TG-3 protein level was increased after receiving atropine. ß-tubulin was used as a loading control.

**Figure 6:**
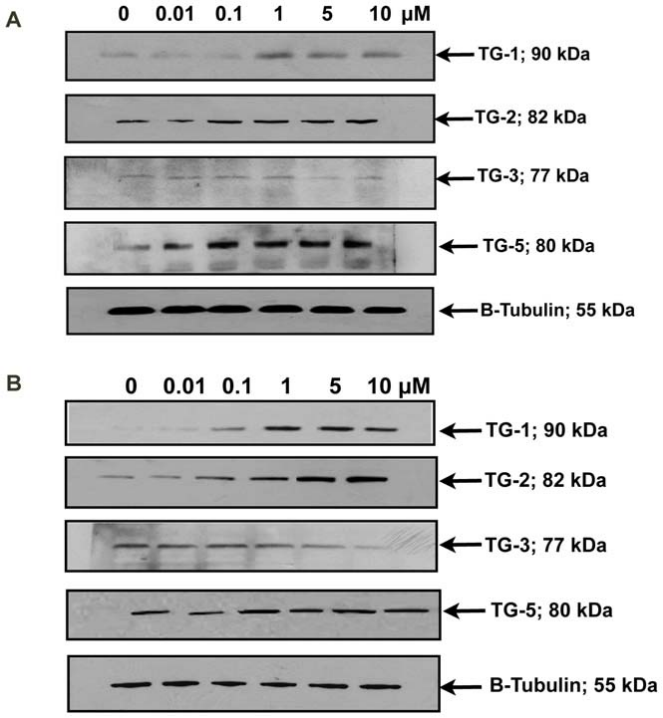
Effect of carbachol treatment on TGs in mouse and human scleral fibroblasts. (A) Western blot image detecting TG proteins in mouse scleral fibroblasts after carbachol treatment. P2 cultured SFs were treated with carbachol at different concentrations for 5 days. Following 5 days of treatment, the total cellular protein was extracted from these cells and TGs proteins detected via Western blot analysis. After carbachol treatment in both SFs, the TGs-1, 2 and 5 protein levels were increased. However, TG-3 protein level was decreased after receiving carbachol. ß-tubulin was used as a loading control. (B) Western Blot image detecting TG proteins in human scleral fibroblasts after carbachol treatment. P2 cultured human scleral fibroblasts were treated with carbachol at concentrations of 0.01 µM, 0.1 µM, 1 µM, 5 µM and 10 µM for 5 days. Following 5 days of treatment, the total cellular protein was extracted from these cells and TG proteins detected via Western blot analysis. It can be observed that the TG-1, TG-2 and TG-5 protein levels were increased after carbachol treatment. However, TG-3 protein level was decreased after receiving carbachol. ß-tubulin was used as a loading control.

The mouse and human SFs were treated with atropine or carbachol at 0.01, 0.1, 1, 5 and 10 µM concentrations for 5 days. Following 5 days of treatment, the total cellular protein was extracted from these cells and TG proteins detected via Western blot analysis. After atropine treatment in both mouse and human SFs, the TGs-1, 2 and 5 protein levels were reduced (Figure 5A and 5B respectively) and the converse was true with carbachol treatment (Figure 6A and 6B respectively). However, TG-3 protein level was increased in both mouse and human SFs after receiving atropine (Figure 5A and 5B respectively) and the converse observed with carbachol (Figure 6A and 6B respectively). These results were similar to changes in transcript levels.

### Transglutaminase-2 activity

**Figure 7:**
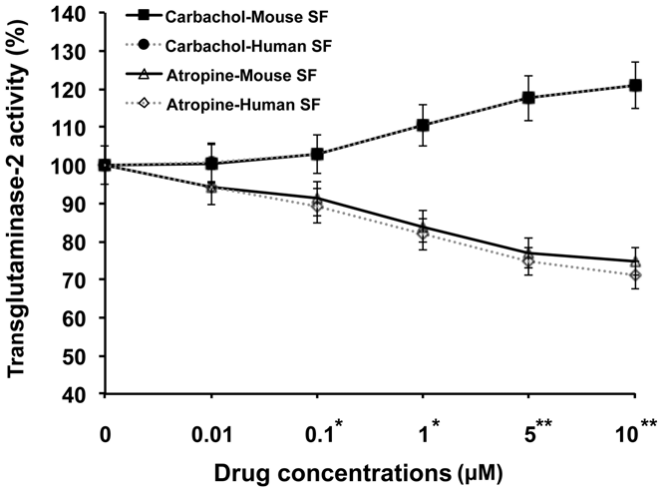
Transamidase activity of TG-2 with stimulation of atropine and carbachol in scleral fibroblasts. Line graph illustrating the results of the transamidase activity of TG-2 in 100 µg of protein lysate from mouse and human cultured scleral fibroblasts. Values were normalized against control values. The mouse and human scleral fibroblasts treated with carbachol and atropine at 0.01, 0.1, 1, 5 and 10 µM for 5 days. The SF treated with carbachol increased the transamidase activity of endogenous cellular TG-2 but this activity was reduced by atropine treatment in a concentration-dependent manner. The values represented the means of three independent samples and error bars represent standard deviation, n = 3, *p<0.05, **p<0.01. Straight lines represent the mouse SF and dashed lines represent the human SF. Open markers are atropine treated and closed markers are carbachol treated SF cells.

The TG-2 colorimetric assay serves as a high throughput and quantitative assay to assess transamidase activity. The mouse SF treated with carbachol and atropine at 0.01, 0.1, 1, 5 and 10 µM for 5 days. The mouse and human SFs treated with carbachol increased the transamidase activity (Figure 7) of endogenous cellular TG-2 but this activity was reduced by atropine treatment in a concentration-dependent manner.

### Quantification of TGs mRNA expression level

**Figure 8:**
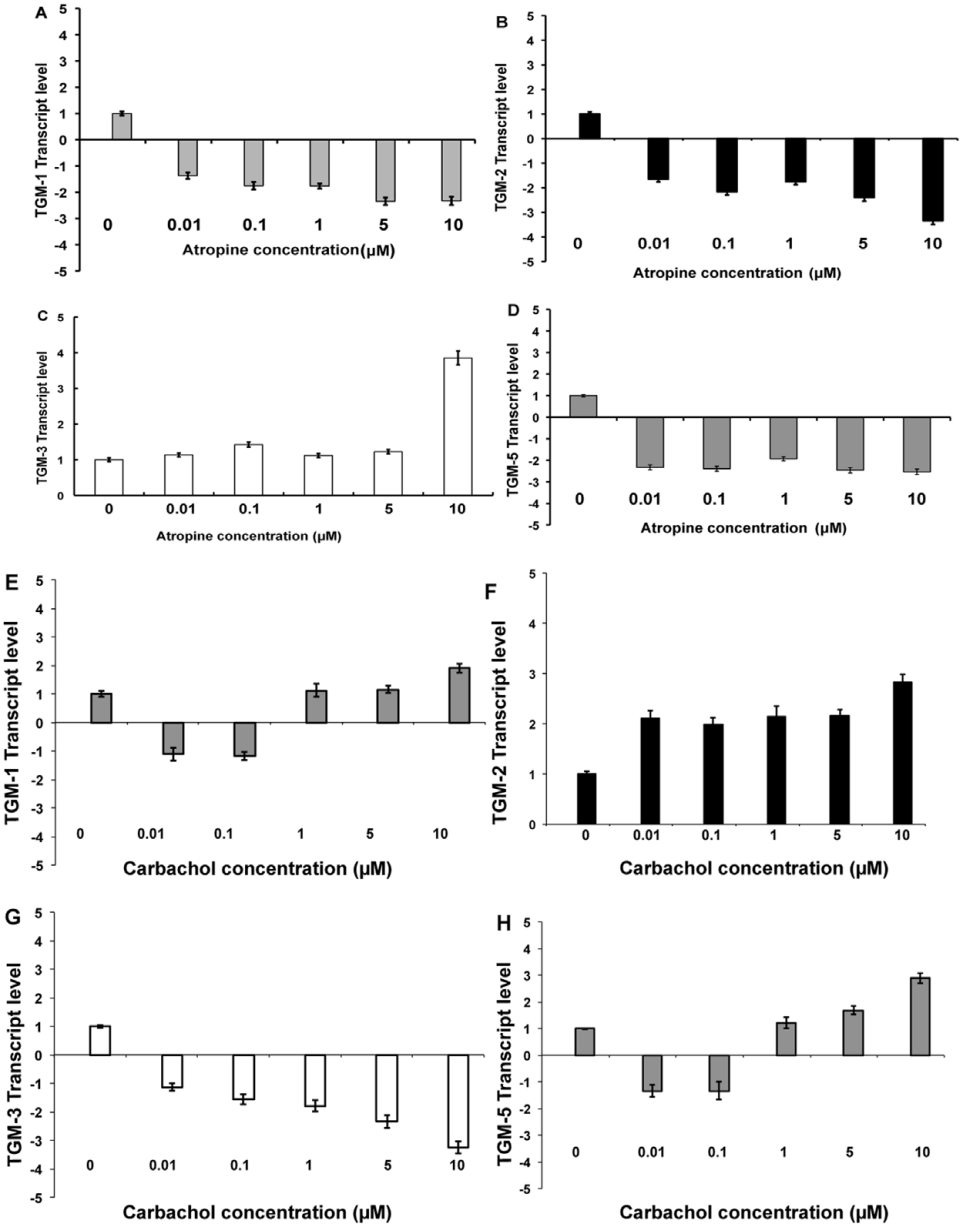
Effect of carbachol on TGM mRNA expression in mouse scleral fibrobalsts. Bar chart illustrating the results of TGM mRNA expression levels of mouse scleral fibroblasts following with stimulation of carbachol. P2 cultured mouse scleral fibroblasts were treated with atropine or carbachol at different concentrations of 0.01 µM, 0.1 µM, 1 µM, 5 µM and 10 µM for 5 days. Following 5 days of treatment, the total RNA was extracted from these cells and TGM transcript level was quantified via qPCR analysis. Height of bars show the means of three independent samples and error bars represent standard deviation. Values are normalized against GAPDH house keeping genes. The TGM-1, 2 and 5 transcript levels were down regulated after receiving atropine (A, B and D respectively) at all concentrations. However, TGM-3 transcript was upregulated in the atropine treated cells (C). The opposite findings were observed with carbachol treatment in both TGM-2 (E) and TGM-3 (F) at all concentrations. However, TGM-1 and 5 were increased only by relatively higher concentrations of carbachol (G and H respectively).

**Figure 9:**
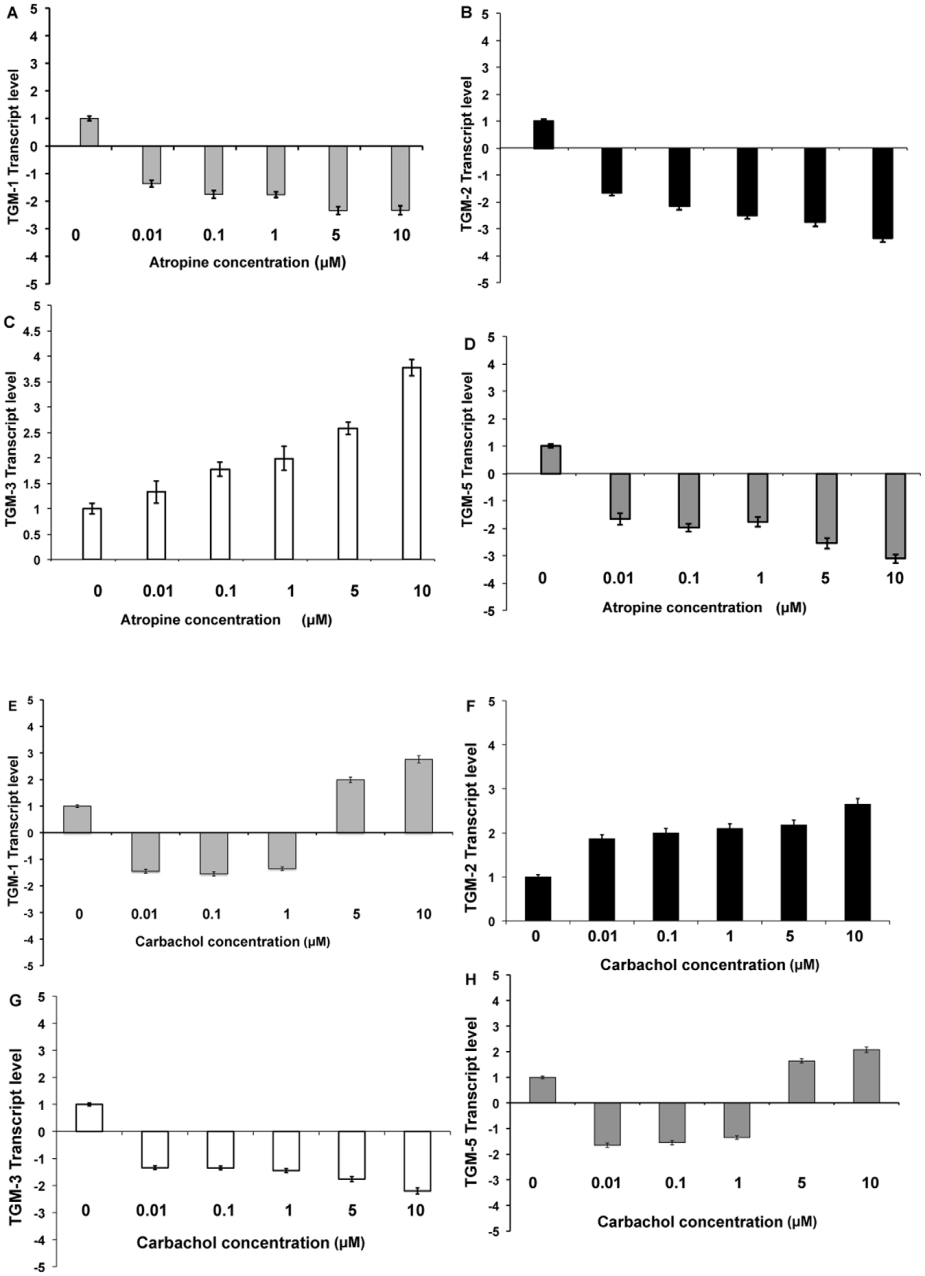
Effect of atropine on TGM mRNA expression in human scleral fibroblasts. P2 cultured human SFs were treated with atropine at different concentrations for 5 days. Following 5 days of treatment, the total RNA was extracted from these cells and TGM transcript level was quantified via qPCR analysis. Height of bars show the means of three independent samples and error bars represent standard deviation. Values are normalized against GAPDH house keeping genes. The TGM-1, 2 and 5 transcript levels were down regulated after receiving atropine (A, B and D respectively) at all concentrations. However, TGM-3 transcript was upregulated in the atropine treated cells (C). The opposite findings were observed with carbachol treatment in both TGM-2 (E) and TGM-3 (F) at all concentrations. However, TGM-1 and 5 were increased only by relatively higher concentrations of carbachol (G and H respectively).

**Figure 10:**
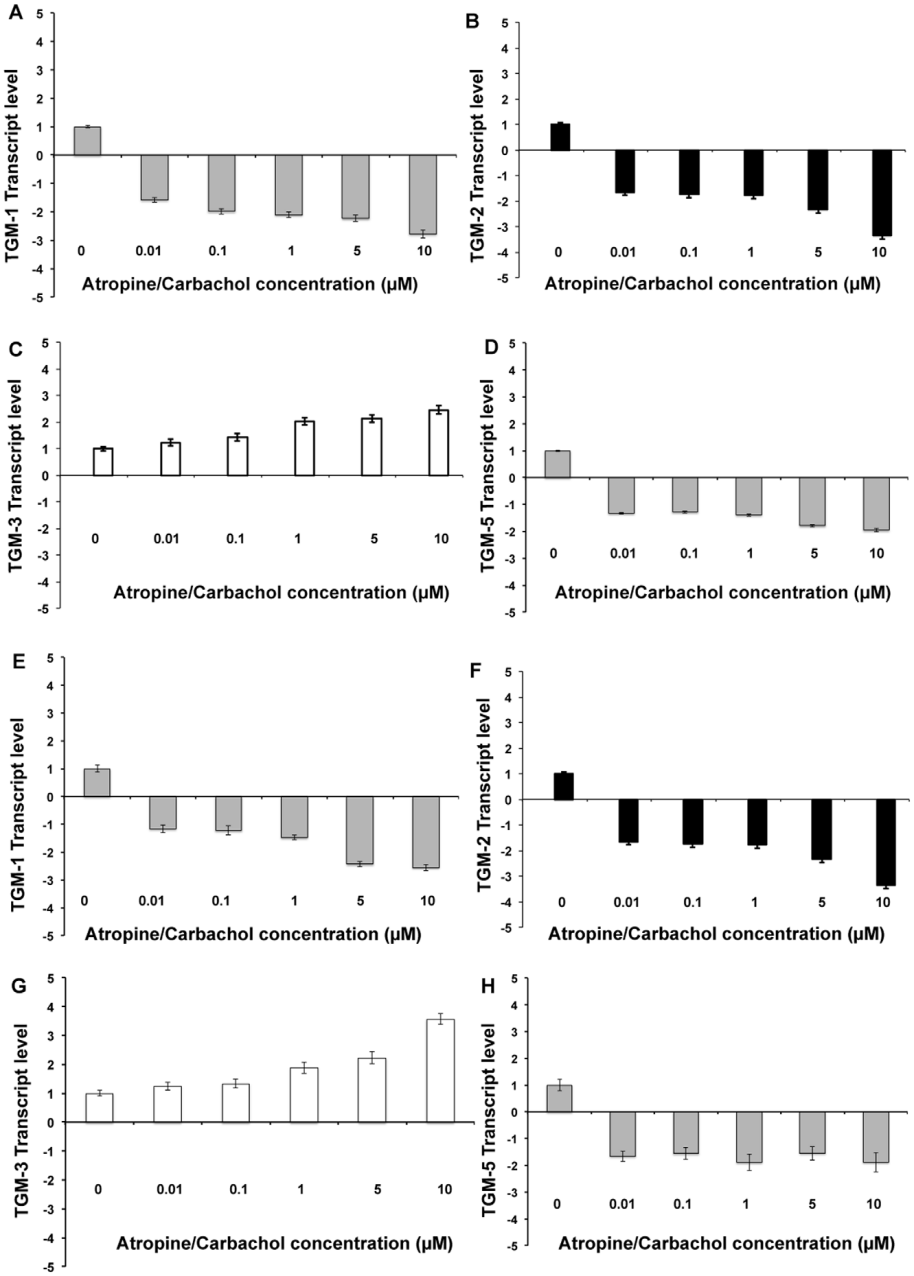
Effect of atropine and carbachol on TGM mRNA expression. Bar chart illustrating the results of TGM mRNA expression levels of mouse and human scleral fibroblasts (A–D and E–H respectively) with atropine and carbachol. P2 cultured mouse (A–D) and human SFs (E–H) was pre-treated with atropine (0.01, 0.1, 1, 5 and 10 µM) 4 hours before being incubated with equimolar carbachol. Following 5 days of treatment, the total RNA was extracted from these cells and TGM transcript level was quantified via qPCR analysis. Height of bars show the means of three independent samples and error bars represent standard deviation. Values are normalized against GAPDH house keeping genes. Atropine abrogated the carbachol-induced activation of TGM-1,2,3,5 in a dose-dependent manner.

P2 cultured mouse and human SFs were treated with atropine or carbachol at different concentrations for 5 days. Following 5 days of treatment, the total RNA was extracted from these cells and TGs transcript level was quantified via real-time qPCR analysis. The TGs-1, 2 and 5 transcript levels were down regulated after receiving atropine in both mouse and human SF at all concentrations (Figures 8A and 8B respectively). However, TG-3 transcript was up regulated in the atropine treated cells. The opposite findings were observed with carbachol treatment in both cells for TG-2 and TG-3 at all concentrations. However, TGs-1 and 5 were increased only by relatively higher concentrations of carbachol (Figures 9A and 9B respectively). Atropine abrogated the carbachol-induced activation of TGs-1,2,3,5 in a dose-dependent manner when mouse and human SFs (Figure 10A–H respectively) were pre-treated with atropine 4 hours before being incubated with equimolar carbachol.

## Discussion

In our study, we found that distinct subtypes of the TGs displayed differential expression in various eye tissues. The localization and expression of TG-2 vary within ocular tissues. TG-2 expression was in the basal layer of cornea and conjunctival epithelium, the basal lamina and in the stroma of these tissues. However, TG-1 and 3 were expressed in the entire thickness of cornea and conjunctival epithelium. The expression of TG-2 in the cornea is different from the other 3 TGs. Components of epithelial basement membranes and dermo-epidermal complexes are substrates of TGs [1]. In cornea, TG-2 is mostly expressed in the epithelial basement membranes. This explains that TG-2 could interact with the hemidesmosomal proteins integrin α_6_β_4_, BP180/collagen XVII, the microfibril components fibrillin 1 and microfibril-associated glycoprotein precursor (MAPG1), which may be cross linked by TG-2 transamidation activity or may be involved in alternate splicing. Hence, key molecules that can regulate extracellular matrix and modulate wound healing might be modified by TG-2 in cornea.

TG-5 expression was not found in the mouse conjunctiva and expressed in the whole corneal epithelium, whereas TG-2 expression was relatively weak in the meibomian glands, compared to other TGs. Immunohistochemical staining showed all 4 TGs to be distributed evenly throughout the sclera. As immunolabelling is limited between the collagen fibre bundles, it is likely that the TGs are localized to the scleral fibroblast processes. This was confirmed by the presence of all 4 TGs in cultured scleral fibroblasts. To the best of our knowledge this appears to be the first report characterizing distributions of TGs in the mouse ocular tissues and in the human sclera.

Previous studies have reported that TG-2 was associated with epithelia and particularly with connective tissue of human and monkey cornea. TG-1 was restricted to the corneal epithelium and TG-3 was absent; however TG-3 was present in the cytoplasm of the granular layer cells of human skin [24]. The distribution of the TGs reported here was different from our results for the mouse eye tissues. These observations may represent real differences in the distribution of corneal TGs between species or alternatively, differences in antibody specificities.

In the monkey cornea and conjunctiva, TG activity can be detected in the intercellular spaces, along the basement membranes, the cytoplasm of the epithelial cells, the superficial stromal keratocytes, as well as in the walls of the conjunctival stromal vessels [24]. Although TG-2 can be found within various ocular cell types, TG-1 was identified only in the corneal epithelium, in the suprabasal cells. In our current study we have carried out TGs expression studies in the SF cells and we would be exploring this further using wound healing models in animals.

A study in rats [25] and other studies have confirmed the role of retinoic acid in the expression of TG-1 [26] and TG-2 [27]. Because TG-1 has a role in ocular surface keratinization, some studies have focused on other upstream triggers of TG-1 including interferon gamma (IFN-γ) [28]. IFN-γ was found to be involved in the ocular surface keratinization of patients with Sjogren's syndrome [29], supporting the importance of the IFN-γ-TG-1 pathway in the ocular surface pathology. TG-2, a multi-functional tissue enzyme of the transglutaminase family, plays a central role in wound healing, apoptosis and ECM production [1]. The kinetics and distribution of TG-2 in the ocular surface were similar to that in dermal wound healing. Early in the healing process, the TG-2 elevation was noted in migrating keratinocytes and infiltrating macrophages, and later, limited to the dermoepidermal junction [5], [6]. This similarity in the wound healing between the ocular surface and skin is important because a large body of literature exists on the role of TG in the *in-vivo* wound healing of the skin, such as those after ultraviolet radiation [30], [31].

In various studies, TG-2 has also been implicated in the signaling pathways of extracellular matrix molecules such as fibronectin and collagen subtypes [32]. Cell adhesion and cell spreading are integral functions regulated by TGs. Primary fibroblasts from TG-2 knock out mice have decreased adherence to culture vessels [33]. Cell-matrix interactions are critical for spreading and migration of cells, as well as the organization of extracellular matrix [34]. In the cornea epithelium for example, TG is involved in the regulation of cornified envelope proteins and apoptosis [35]. In the rat cornea [25], TG-2 was strongly up regulated in the early wound healing process and actively migrating cornea epithelial cells were expressing TG-2.

Anti-muscarinic drugs were able to change collagen and other structural molecules in animal models of myopia [36], [37], establishing a link to scleral connective tissue in these pathways. However, even though muscarinic receptor subtypes may be involved in scleral remodeling, we do not know the intermediate and downstream target molecules. Our present study that showed atropine decreased TGs-1, 2 and 5 proteins and carbachol increased. However, for unknown reasons, these drugs have the opposite effect on TG-3 protein expression. Moreover, atropine abrogated the carbachol-induced activation of SF in a dose-dependent manner.

In fibroblasts cultured from scleral explants, treatment with atropine induced a reduction in the level of TG-2 protein and mRNA. Previous investigation showed that TG activity increased after agonistic stimulation [19]. This suggests that manipulation of TG through muscarinic receptor may be a plausible method of intervention in scleral remodeling and human diseases such as myopia, which involved scleral remodeling [37].

In the eye, researchers are beginning to appreciate the importance of TGs in the ocular surface, anterior and posterior segment diseases [38], [39]. Taking advantage of the understanding of TGs in critical processes such as wound healing and modulation of inflammation, translational research in ocular diseases will be greatly enhanced, including ocular surface diseases, glaucoma, cataract and proliferative vitreoretinopathy. It is important to know that drugs have different effects on different TGs, because some functions are more sub-served by one TG than another. Kalinin et al [40] reported that cornified envelope assembly occurs by passing substrates from TG-5 to TG-3 to TG-1, so it may be possible to manipulate specific TG and not others. However, in view of functional redundancy, what is not clear is how different TGs can compensate for one another since all of them have transamidase activity. In addition, only TG-2 has G protein activity, G protein function may be a preferred treatment target in wound healing and not the transamidase.

In conclusion, our results confirm that TGs-1, 2, 3 and 5 are expressed in ocular tissues and associated glands. Real-time qPCR and Western blot results showed that muscarinic antagonist down-regulates TGs-1, 2 and 5 in both cultured mouse and human SFs. However, TG-3 was up regulated in the atropine treated cells. The opposite findings were observed with carbachol treatment in both cells for TGs-1, 2, 5 and 3. Atropine abrogated the carbachol-induced activation of SF in a dose-dependent manner. These results suggest that manipulation of TGs by way of muscarinic receptor acting drugs may be a plausible method of intervention in wound healing and scleral remodeling.
